# Highly Sensitive Temperature Sensor Based on Cascaded Polymer-Infiltrated Fiber Mach–Zehnder Interferometers Operating near the Dispersion Turning Point

**DOI:** 10.3390/polym14173617

**Published:** 2022-09-01

**Authors:** Jia He, Fengchan Zhang, Xizhen Xu, Bin Du, Jiafeng Wu, Zhuoda Li, Zhiyong Bai, Jinchuan Guo, Yiping Wang, Jun He

**Affiliations:** 1 Key Laboratory of Optoelectronic Devices and Systems of Ministry of Education/Guangdong Province, College of Physics and Optoelectronic Engineering, Shenzhen University, Shenzhen 518060, China; 2 Shenzhen Key Laboratory of Photonic Devices and Sensing Systems for Internet of Things, Guangdong and Hong Kong Joint Research Centre for Optical Fibre Sensors, Shenzhen University, Shenzhen 518060, China

**Keywords:** optical fiber sensor, fiber interferometer, femtosecond laser micromachining, dispersion turning point, temperature measurement

## Abstract

High-accuracy temperature measurement plays a vital role in biomedical, oceanographic, and photovoltaic industries. Here, a highly sensitive temperature sensor is proposed and demonstrated based on cascaded polymer-infiltrated Mach–Zehnder interferometers (MZIs), operating near the dispersion turning point. The MZI was constructed by splicing a half-pitch graded index fiber (GIF) and two sections of single-mode fiber and creating an inner air cavity based on femtosecond laser micromachining. The UV-curable polymer-infiltrated air cavity functioned as one of the interference arms of MZI, and the residual GIF core functioned as the other. Two MZIs with different cavity lengths and infiltrated with the UV-curable polymers, having the refractive indexes on the different sides of the turning point, were created. Moreover, the effects of the length and the bending way of transmission SMF between the first and the second MZI were studied. As a result, the cascaded MZI temperature sensor exhibits a greatly enhanced temperature sensitivity of −24.86 nm/°C based on wavelength differential detection. The aforementioned result makes it promising for high-accuracy temperature measurements in biomedical, oceanographic, and photovoltaic applications.

## 1. Introduction

Temperature is one of the most common indicators used for condition monitoring in biomedical, oceanographic, and photovoltaic industries [1,2,3,4]. There is a growing demand for high-accuracy temperature sensors to investigate the process of dynamic change in these cases. For example, the monitoring of the brain temperature is essential for the therapy for patients with ischemic stroke [1]. Ocean temperature variation is an important basic parameter for the human exploration of the ocean and provides necessary information to investigate global climate changes [2]. In situ and continuous monitoring of the chemical and thermal state of a cell during operation is crucial to ensure its safety [3,4]. Moreover, it is beneficial for achieving the aforementioned high-accuracy temperature measurements by using high-sensitivity temperature sensors, and hence they have attracted more and more attention. The main types of commercial temperature sensors include thermocouples [5], temperature-sensitive paint [6], and infrared thermal imagers [7,8]. However, such temperature sensors have their own shortcomings. For example, they usually have large sizes and are mainly focused on single-point temperature monitoring. Optical fiber-based devices are more attractive for temperature sensing, owing to their compact size, the capability of multiplexing, and immunity to electromagnetic interference.

Fiber Bragg grating sensors are the most typical fiber-optic temperature sensors; however, the temperature sensitivity of them is usually very low (~10 pm/°C) [9]. To date, Mach–Zehnder interferometers based on various in-fiber microstructures (i.e., internal air cavities [10], tapers [11,12], and offset splicing joints [13]) have been proposed to achieve a higher temperature sensitivity of ~2 nm/°C. However, it is still limited by the low thermo-optic coefficient (TOC) of silica (i.e., ~8.3 × 10^−6^ RIU/°C) [14]. Hence, the fiber interferometer infiltrated or covered with materials having large TOCs is considered a better alternative for high-accuracy temperature measurements. For example, Shi et al. reported a photonic crystal fiber (PCF) Sagnac interferometer by infiltrating all air holes in PCF with ethanol and obtained a high temperature sensitivity of 16.81 nm/°C [15]. Very recently, Cheng et al. proposed a tapered multicore fiber MZI interferometer covered with PDMS and achieved a high temperature sensitivity of 25 nm/°C [16]. Additionally, a temperature sensitivity of up to −84.72 nm/°C can be obtained by employing an MZI temperature sensor based on a D-shaped cavity, which is filled with liquid [17]. However, the aforementioned temperature sensors have typically poor thermal repeatability and stability due to the volatility and fluidity of thermal-sensitive liquid material.

In this paper, we propose and demonstrate a highly sensitive temperature sensor by cascading two UV-curable polymer-infiltrated Mach–Zehnder interferometers (MZIs) operating near the dispersion turning point (DTP). The single MZI consists of two single-mode fiber (SMF) pigtails, a half-pitch graded index fiber (GIF) that serves as an in-fiber collimator, and an inner air cavity created by femtosecond laser micromachining. A cascaded MZI temperature sensor with cavity lengths of 60 and 80 μm in the first and the second MZI was created. Moreover, two types of UV-curable polymers with the refractive indexes of 1.460 and 1.540 were employed to infiltrate these two MZIs, respectively. The UV-curable polymer-infiltrated air cavity functioned as one of the interference arms of MZI and the residual GIF core functioned as the other. Moreover, the effects of the length and the bending way of transmission SMF between the first and the second MZI were studied. The temperature response results showed that such a cascaded MZI temperature sensor exhibited a greatly enhanced temperature sensitivity of −24.86 nm/°C based on wavelength differential detection. The aforementioned result makes it promising for high-accuracy temperature measurements in biomedical, oceanographic, and photovoltaic applications.

## 2. Materials and Methods

**Figure 1:**
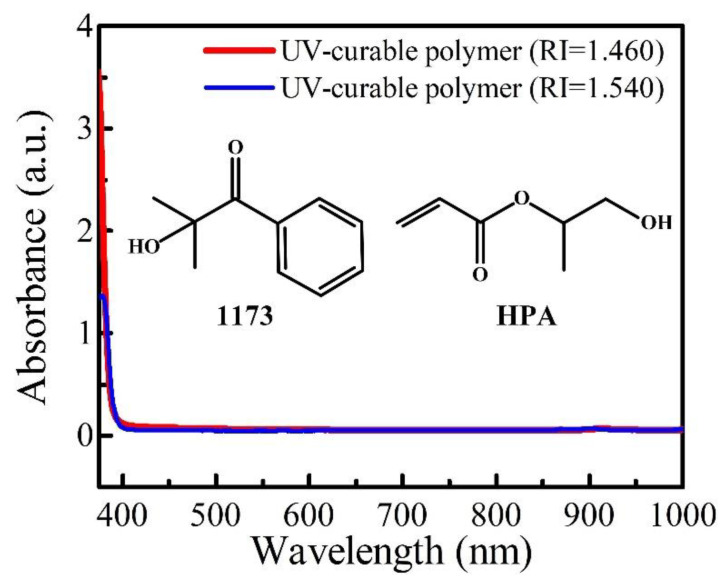
Absorption spectra of the two types of UV-curable polymers used in this work.

Generally speaking, refractive index (RI)-matching liquid is the typical thermo-sensitive liquid material for the infiltration of in-fiber microstructures to obtain highly sensitive MZI temperature sensors [16]. However, such a kind of thermo-sensitive liquid material is not suitable for fabricating temperature sensors with great thermal stability and repeatability, thus UV-curable polymer with better mechanical property has been investigated. The UV-curable polymer (RI = 1.460) used in this work consists of photoinitiator 1173 (2-Hydroxy-2-Methyl-1-Phenyl-1-Propanone, as shown in Figure 1) with a mole ratio of 7–15%, oligomer (Acrylated Aliphatic Urethane) with a mole ratio of 45–55%, and acrylate monomer (Hydroxypropyl Acrylate, HPA, as shown in Figure 1) with a mole ratio of 40–50%. The UV-curable polymer (RI = 1.540) used in this work consists of oligomer (Pentaerythritol Tetra(3-mercaptopropionate)) with a mole ratio of 30–50% and acrylate monomer (Hydroxypropyl Acrylate, HPA) with a mole ratio of 36–60%. The absorption spectra of the two types of UV-curable polymers used in this work were studied by using a UV-Vis spectrofluorometer (METASH, UV8000), both showing a very high transmittance in the visible and infrared wavelength range in Figure 1. Compared with other conventional polymers, as shown in Table 1, the UV-curable polymer materials used in this work were chosen owing to their superior characteristics, including the above-high light transmittance, their large TOC, controllable RI, simple fabrication process, and low cost.

**Figure 2:**
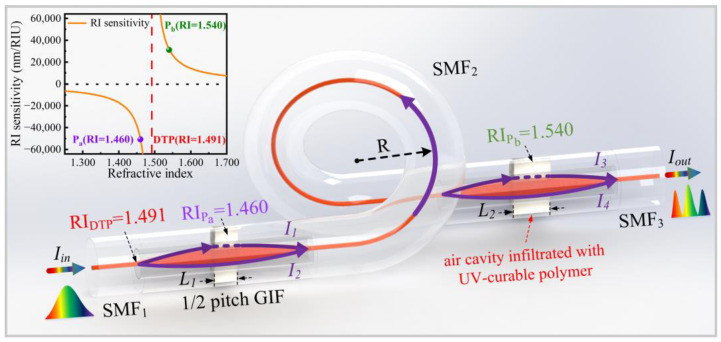
Schematic diagram of the proposed highly sensitive temperature sensor based on cascaded polymer-infiltrated MZIs operating near the DTP. (Inset: the calculated RI sensitivities of the MZI infiltrated with polymers with different RIs).

As illustrated in Figure 2, the proposed highly sensitive temperature sensor consists of two MZI temperature sensors created by femtosecond laser micromachining, infiltrated with the aforementioned UV-curable polymer, and cascaded by splicing together. The working principle of the fabricated MZI temperature sensor is as follows. For example, the two SMF pigtails (i.e., SMF_1_ and SMF_2_) of the first MZI (i.e., MZI_1_) serve as a lead-in and lead-out fiber, respectively. A half-pitch GIF with a length of 490 μm serves as a collimator, which can reduce insertion loss of MZI [23]. The light trajectory in the GIF is quasi-sinusoidal, which implies that the mode area of the GIF reaches its maximum in the quarter-pitch position and its minimum at the end of the GIF. Thus, on one lateral side of the core of GIF in the quarter-pitch position, an inner air cavity was fabricated. As a result, the UV-curable polymer-infiltrated inner air cavity functioned as the sensing arm and the residual GIF core is used as a reference arm. The light intensity in the SMF_2_ can be described as [24]:

(1) $$I=I_{1}+I_{2}+2\sqrt{I_{1}I_{2}}\cos(2\pi L_{1}\Delta n_{1}/\lambda_{s}),\;$$

where I_1_ and I_2_ represent the beam power of the two interference arms, L_1_ is the air cavity length, λ_s_ is the wavelength, Δn_1_ = n_polymer1_ − n_core_ is the RI difference between the UV-curable polymer (n_polymer1_) and GIF core (n_core_ = 1.491), and L_1_Δn_1_ is the optical path difference (OPD) between two interference beams of the MZI_1_. The resonance dip wavelength can be expressed as:

λ_m_ = 2L_1_Δn_1_/(2m + 1), (2)

where m is an integer, and λ_m_ is the wavelength of the m_th_ order interference dip. The free spectral range (FSR) of the interference fringe dip is determined by OPD, as:

FSR = λ_s_^2^/(L_1_Δn_1_). (3)

The working principle of the second MZI (i.e., MZI_2_) is the same as MZI_1_ and λ_L_ is the dip wavelength of MZI_2_. When the transmission light is injected into the MZI_2_, the interference signals are combined into the whole spectrum. Using a curved transmission SMF (i.e., SMF_2_) can further suppress the multimode interference noise. Moreover, the RI sensitivity can be derived from Equation (2) as dλm/d(Δn_1_) = λm/Δn_1_ and the calculated results are shown in the inset of Figure 2. Note that, the RI sensitivity of the UV-curable polymer-infiltrated MZI at the DTP of 1.491 tends to be infinite and is opposite in signs on the different sides of DTP. Furthermore, the temperature sensitivity of MZI can be derived as:

dλ_m_/dT = (λ_m_/Δn_1_(T))·(δ_polymer1_ − δ_core_), (4)

where Δn_1_(T) = n_polymer1_(T) − n_core_(T) is the RI difference between the UV-curable polymer and GIF core under temperature T, δ_polymer1_ is the TOC of UV-curable polymer (~−2 × 10^−4^ RIU/°C) [22] and δ_core_ is the TOC of silica (~8.3 × 10^−6^ RIU/°C) [14]. As a result, the temperature sensitivity is also opposite in signs on the different sides of DTP. Hence, the air cavity lengths of MZI_1_ and MZI_2_ are designed to be 60 μm and 80 μm, respectively. Moreover, the RI of the UV-curable polymers used in MZI_1_ and MZI_2_ are chosen to be 1.460 and 1.540, respectively.

**Figure 3:**
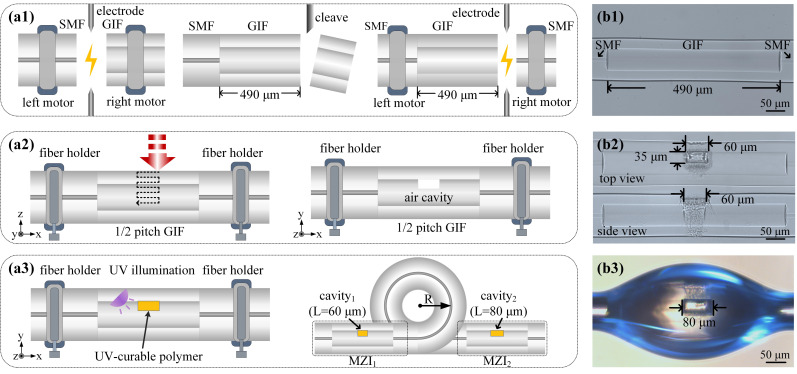
(**a**l–**a**3) Schematic diagram of the fabrication process of a cascaded polymer-infiltrated MZI temperature sensor: (**a**l) in step 1, a half-pitch GIF was spliced to two sections of SMF; (**a**2) in step 2, an inner air cavity was created by femtosecond laser micromachining; (**a**3) in step 3 and step 4, the air cavity was infiltrated with UV-curable polymer and two MZI temperature sensors were cascaded. (**b**1–**b**3) The corresponding microscopic images of a cascaded polymer-infiltrated MZI temperature sensor fabricated after step 1–3, respectively.

Figure 3 illustrates the fabrication process of a cascaded polymer-infiltrated MZI temperature sensor, which involves four steps [25,26]. In step 1, as shown in Figure 3a1, a conventional SMF was spliced to a section of a GIF with a core diameter of 62.5 μm by means of a commercial fusion splicer. The spliced configuration was then cleaved to the length of 490 μm (i.e., half-pitch length) by using the precision cleaving configuration. Then, the well-cleaved end of the GIF was spliced to another SMF. The corresponding microscope image is shown in Figure 3b1. In step 2, as shown in Figure 3a2, a rectangular inner air cavity was obtained in the quarter-pitch position of GIF by femtosecond laser micromachining. The wavelength, pulse width, and repetition of the used femtosecond laser (Spectra-Physics) are 800 nm, 120 fs, and 1 kHz, respectively. An average on-target laser power of 15 mW was applied. The top-view and side-view microscope images of the fabricated MZI_1_ are shown in Figure 3b2. In step 3, as shown in Figure 3a3, the inner air cavity was infiltrated by the UV-curable polymer, and then cured by UV illumination for 1 h. Moreover, thermal annealing at 50 °C for 12 hours was applied, intended to obtain a thermal stable MZI temperature sensor. Figure 3b3 shows the microscope image of the UV-curable polymer-infiltrated MZI_2_. In step 4, as shown in Figure 3a3, the MZI_1_ and MZI_2_ were cascaded by splicing the two SMF pigtails together. Furthermore, the SMF (i.e., SMF_2_, as shown in Figure 2) between MZI_1_ and MZI_2_ was bent. 

## 3. Experiments and Results

**Figure 4:**
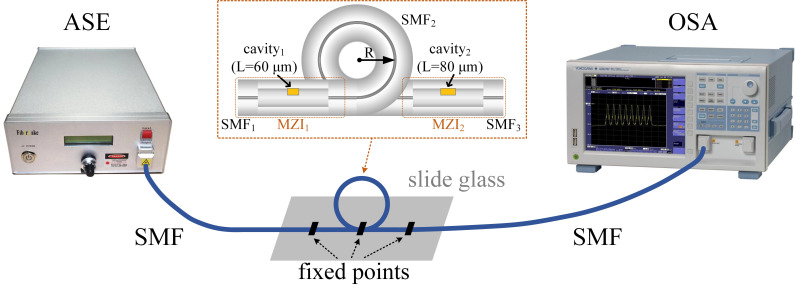
Schematic diagram of the experimental setup for measuring the transmission spectra of the cascaded MZI samples. (ASE: amplified spontaneous emission, OSA: optical spectrum analyzer, inset: schematic diagram of the cascaded MZI samples).

**Figure 5:**
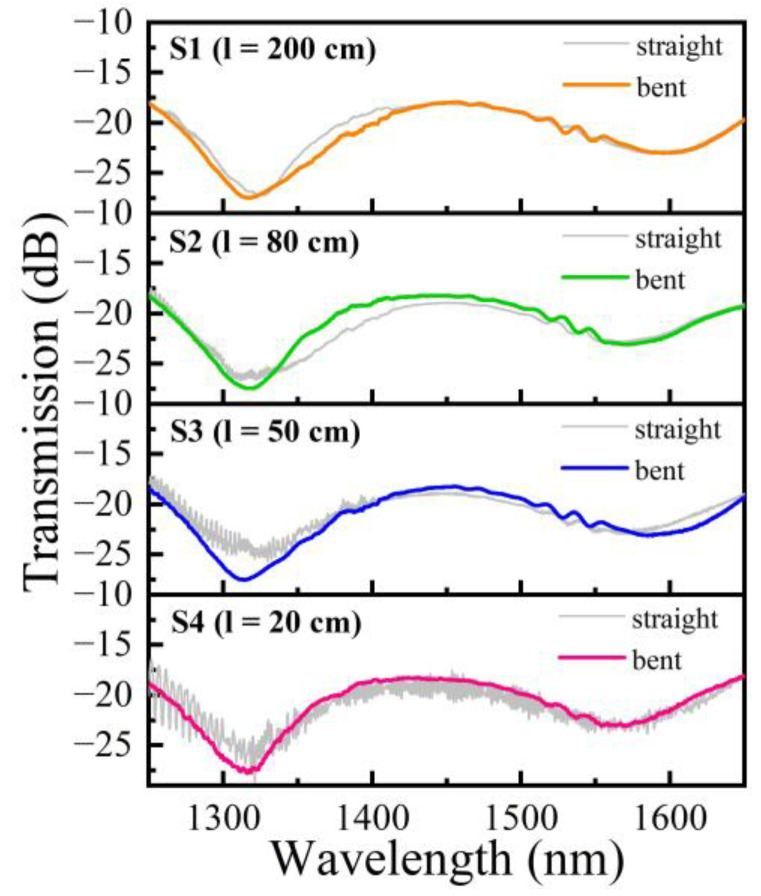
Transmission spectra of four fabricated cascaded MZI samples S1, S2, S3, and S4 with decreasing lengths of transmission SMF (i.e., SMF_2_) between MZI_1_ and MZI_2_ of 200, 80, 50, and 20 cm, respectively, in the straight and bent state.

We fabricated four cascaded MZI samples S1, S2, S3, and S4 with decreasing lengths of transmission SMF (i.e., SMF_2_) between MZI_1_ and MZI_2_ of 200, 80, 50, and 20 cm, respectively. The effect of air cavity lengths on the performance of MZIs has been well studied in the previous work [25]. Taking the factors (including a smaller FSR, an acceptable insertion loss, and the measurement range of the optical spectrum analyzer) and the results deduced from Equation (3) into consideration, the air cavity lengths of MZI_1_ and MZI_2_ of these cascaded MZI samples are designed to be 60 μm and 80 μm, respectively. As shown in Figure 4, the transmission spectra of the cascaded MZI samples were investigated based on an amplified spontaneous emission (ASE) light source (FiberLake) and an optical spectrum analyzer (OSA, YOKOGAMA). The cascaded MZI sample was fixed onto a slide glass by employing UV-curable polymer on the two leading fibers and SMF_2_ (as shown in the inset of Figure 4). Note that the light directed into MZI_1_ excites several modes. A part of light transmits into the core of SMF_2_ as the fundamental mode, while another part propagates in the cladding as the high order mode. When the transmission light is directed to MZI_2_, multimode interference noise is excited. As shown in Figure 5, the multimode interference noise in the spectrum increases as the length of SMF_2_ decreases, and relatively smooth spectrum can be obtained when SMF_2_ was in the bent state, whatever the length of it is. Moreover, to reduce the temperature measurement errors, the length of SMF_2_ was set to be 20 cm ultimately.

**Figure 6:**
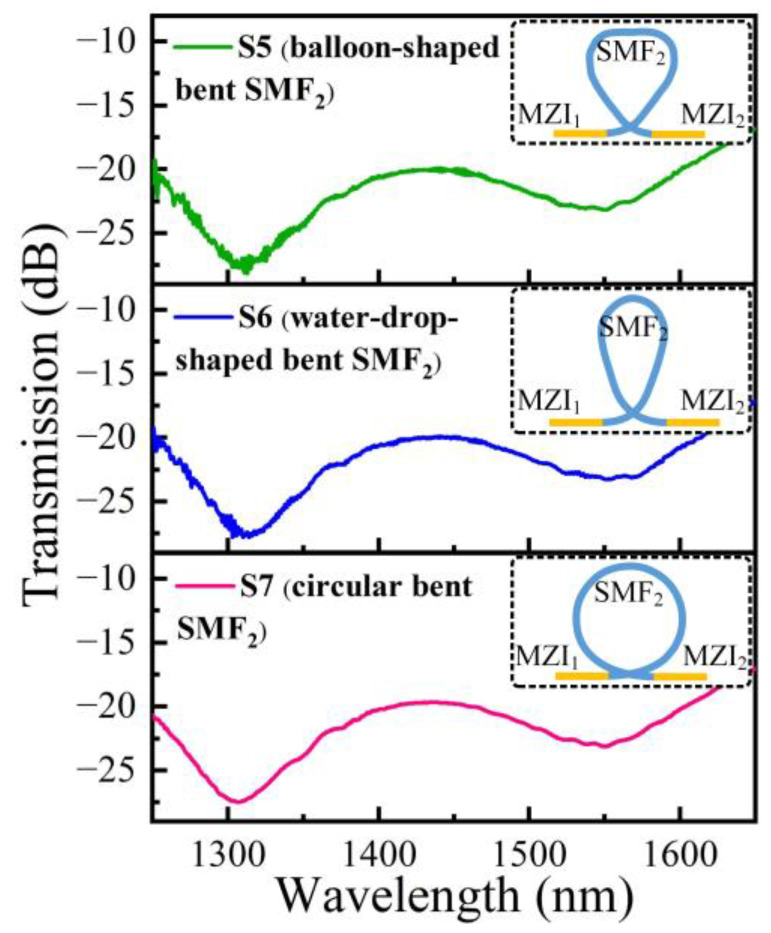
Transmission spectra of three fabricated cascaded MZI samples S5, S6, and S7 with varying bending ways of 20 cm long transmission SMF (i.e., SMF_2_) between MZI_1_ and MZI_2_.

Furthermore, we investigated the cascaded MZI samples S5, S6, and S7 obtained with varying bending ways of 20 cm long SMF_2_. The bent SMF_2_ serves as a transmission fiber and a mode stripper, having the ability to strip the high-order modes effectively due to their large bend losses [27]. As shown in Figure 6, there are still large multimode interference noises in the spectra of the two samples with balloon-shaped bent SMF_2_ and water-drop-shaped bent SMF_2_, respectively. The result indicates that when the SMF_2_ was in the circular bent state with a radius of ~3.2 cm, the high-order modes in SMF_2_ exhibited larger bend losses compared to other bending ways, and hence the multimode interference noises in the spectrum of cascaded MZI temperature sensor can be suppressed effectively.

**Figure 7:**
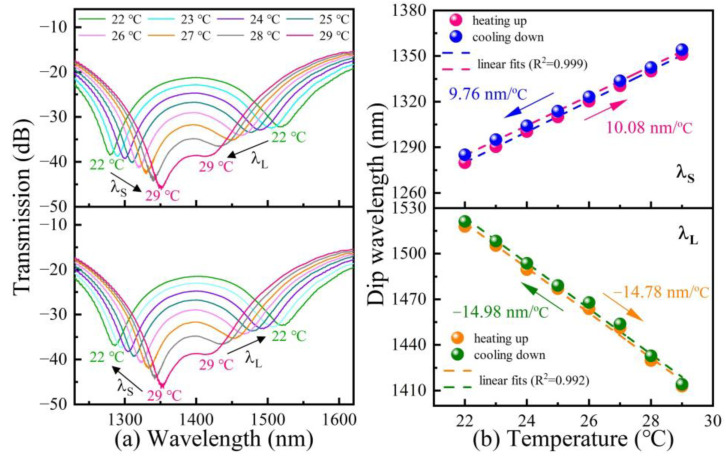
(**a**) Transmission spectra evolutions of the cascaded MZI temperature sensor in the case of temperature cycling from 22 to 29 °C; (**b**) temperature response of the dip wavelengths λ_S_ and λ_L_ in the transmission spectrum of the cascaded MZI temperature sensor.

Subsequently, we investigated the temperature response of a cascaded MZI temperature sensor by putting it into a high-precision oven (accuracy: up to 0.1 °C). The temperature was varied from 22 to 29 °C and was maintained for 20 min at each measurement point. As shown in Figure 7a, it is obvious that λ_S_ exhibits a ‘red’ shift with increasing temperature and a ‘blue’ shift with decreasing temperature. It can be seen from Figure 7b that the temperature sensitivities of λ_S_ are 10.08 nm/°C and 9.76 nm/°C in the heating and cooling processes, respectively. However, the evolution of the dip wavelength λ_L_ is in contrast and the temperature sensitivities are −14.78 nm/°C and −14.98 nm/°C, respectively. It can be seen from Equation (4) that the positive temperature sensitivity of MZI_1_ results from the RI difference Δn_1_ < 0, and the TOC of the UV-curable polymer is negative (~−2 × 10^−4^ RIU/°C) [22]. In contrast, the negative temperature sensitivity of MZI_2_ results from the RI difference Δn_2_ > 0.

**Figure 8:**
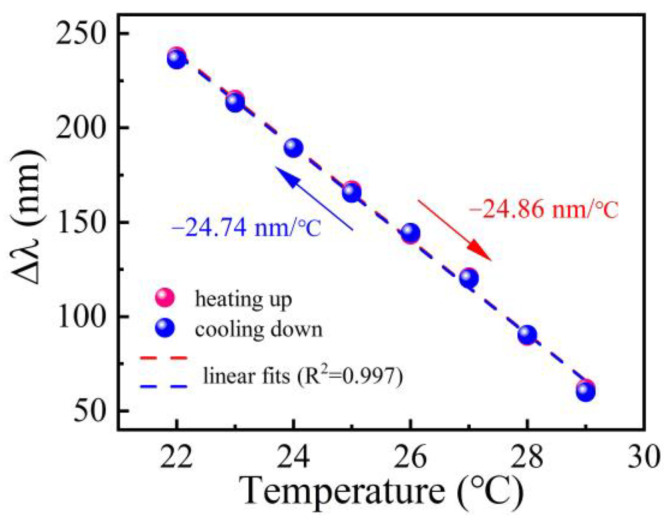
Differential wavelength (i.e., λ_L_−λ_S_) as a function of the temperature in the case of temperature cycling from 22 to 29 °C.

Figure 8 shows the differential wavelength (i.e., λ_L_ − λ_S_) of the cascaded MZI temperature sensor as a function of the temperature in the case of temperature cycling from 22 to 29 °C. It can be seen that the data could be well fitted by linear functions. It is obvious that the cascaded MZI temperature sensor exhibits greatly enhanced temperature sensitivities of −24.86 nm/°C and −24.74 nm/°C in the heating and cooling processes, respectively, based on wavelength differential detection. The temperature sensitivities are almost twice that of a single MZI temperature sensor [25,26]. Hence, a highly sensitive MZI temperature sensor could be obtained based on cascading and wavelength differential detection. Moreover, it should be noted that a narrow temperature range of 22–29 °C was performed in the experiment. However, the operating temperature range of the proposed cascaded MZI temperature sensor could be further extended to a wider range from −80 to 70 °C by using a broadband OSA or an intensity demodulation method [25].

## 4. Conclusions

We have proposed and demonstrated a highly sensitive temperature sensor by cascading two UV-curable polymer-infiltrated MZIs operating near the DTP. The effects of the length and the bending way of transmission SMF between the first and the second MZI were studied. After optimizing these parameters, a cascaded MZI temperature sensor without multimode interference noises in the transmission spectrum was obtained. The temperature test showed such a cascaded MZI temperature sensor has a greatly enhanced temperature sensitivity of −24.86 nm/°C, based on wavelength differential detection. Hence, the simple fabrication process, compact size, and ultrahigh temperature sensitivity of the proposed cascaded MZI-based temperature sensor would make it eminently suitable for utilization for high-accuracy temperature measurements in biomedical, oceanographic, and photovoltaic applications.

## Figures and Tables

**Table 1:** Properties of different types of polymers.

Polymer	TOC (RIU/°C)	Refractive Index	Reference
PMMA	−1.3 × 10^−4^	1.48	[18,19]
PC	−0.9 × 10^−4^	1.585	[20]
Silicone	−1.3 × 10^−4^	1.492	[21]
The UV-curable polymers used in this work	−2 × 10^−4^	Could be controlled by changing the density	[22]

## Data Availability

The data presented in this study are available on request from the corresponding author.
